# Trends in serum creatinine testing in Oxfordshire, UK, 1993–2013: a population-based cohort study

**DOI:** 10.1136/bmjopen-2015-009459

**Published:** 2015-12-16

**Authors:** Jason Oke, Brian Shine, Emily McFadden, Richard Stevens, Daniel Lasserson, Rafael Perera

**Affiliations:** 1 Nuffield Department of Primary Care Health Sciences, University of Oxford, Oxford, UK; 2 Department of Clinical Biochemistry, John Radcliffe Hospital, University of Oxford, Oxford, UK; 3 NIHR Oxford Biomedical Research Centre, John Radcliffe Hospital, Oxford, UK

**Keywords:** PRIMARY CARE, EPIDEMIOLOGY, Chronic kidney disease, serum creatinine

## Abstract

**Objectives:**

To determine how many kidney function tests are done, on whom, how frequently they are performed and how they have changed over time.

**Design:**

Retrospective study of all serum creatinine, urine albumin and urine creatinine tests.

**Setting:**

Primary and secondary care in Oxfordshire from 1993 to 2013.

**Participants:**

Unselected population of 1 220 447 people.

**Main outcome measures:**

The total number of creatinine and urinary protein tests ordered from primary and secondary care and the number of tests per year stratified by categories of estimated glomerular filtration rate (eGFR). The frequency of testing in patients having their kidney function monitored.

**Results:**

Creatinine requests from primary care increased steadily from 1997 and exceeded 220 000 requests in 2013. Tests corresponding to normal kidney function (eGFR >60/mL/min/1.73 m^2^) constituted 59% of all kidney function tests in 1993 and accounted for 83% of all tests in 2013. Test corresponding to chronic kidney disease (CKD) stages 3–5 declined after 2007. Reduced kidney function, albuminuria, male gender, diabetes and age were independently associated with more frequent monitoring. For a female patient between 61 and 80 years and with stage 3a CKD, the average number of serum creatinine tests (95% CI) was 3.23/year (3.19 to 3.26) and for a similar woman with diabetes, the average number of tests was 5.50 (5.44 to 5.56) tests per year.

**Conclusions:**

There has been a large increase in the number of kidney function tests over the past two decades. However, we found little evidence that this increase is detecting more CKD. Tests are becoming more frequent in people with and without evidence of renal impairment. Future work using a richer data source could help unravel the underlying reasons for the increased testing and determine how much is necessary and useful.

**Strengths and limitations of this study**

- This study uses 20 years of data from a single laboratory that serves a well-defined population, typical of the wider UK population.
- To our knowledge, no other study has looked specifically at changes in serum creatinine test ordering rates, over time, in the UK.
- We did not have access to data on patient history or prescriptions or reasons for test ordering and so cannot comment on whether they were ordered with appropriate frequency.

## Introduction

Serum creatinine (SCr) is widely used to measure renal function in the detection, diagnosis and management of chronic kidney disease (CKD) and other renal disorders such as acute kidney injury (AKI). Urinary albumin and protein are markers of kidney damage but also indicate disease from anywhere within the urinary tract. As both reduced renal function and elevated urinary albumin or protein are independently associated with adverse kidney outcomes (end-stage renal disease, AKI and progression of CKD)^1^ as well as cardiovascular events in the general population, monitoring may be warranted . Renal function monitoring with SCr testing is also critical for the safe administration of a wide range of therapeutic agents including those for bipolar disorder,^2^ cancer,^3^ hypertension^4^ and diabetes. In order to take into account factors such as age, gender and ethnic group, SCr can be converted to an estimated glomerular filtration rate (eGFR) through equations such as Modification of Diet in Renal Disease (MDRD)^5^ or Chronic Kidney Disease Epidemiology Collaboration (CKD-EPI).^6^

The Kidney Disease Outcomes and Quality Initiative (KDOQI) clinical practice guidelines in early 2002^7^ proposed that stages of CKD be defined primarily according to eGFR. In 2004, the Department of Health's National Service Framework for Renal Services^8^ adopted the KDOQI staging classification of CKD. In the same year, the Quality and Outcomes Framework (QOF), part of the contract for UK general practice (GP),^9^ introduced incentives for the recording of SCr in people with diabetes or on lithium therapy. The 2006/2007 extension of QOF required GP doctors to maintain a register of adults with CKD stages 3–5.^10^ In 2008, guidance from the National Institute for Health and Care Excellence (NICE)^11^ recommended monitoring of renal function through creatinine testing in high-risk groups. The 2009/2010 extension of QOF did not specifically incentivise SCr testing or eGFR calculation, but incentivised the monitoring of urinary markers of kidney disease such as urinary albumin-creatinine ratio (ACR) in patients on the CKD register.^9^

Motivated by a previous analysis of lipid testing in the same region,^12^ we examined SCr tests and urinary albumin and protein tests ordered in Oxfordshire (UK) from 1993 to 2013. Second, we describe the distribution of CKD stages among those tested over time. Lastly, we explore how the frequency of monitoring has varied over time and between patients with different characteristics.

## Methods

Data included all requests for SCr, ACR, protein to creatinine ratio (PCR) and glycated haemoglobin (HbA1c) measurements from the Oxford University Hospitals Trust Clinical Biochemistry laboratories for the entire periods covered by the database (May 1969 to November 2014). One of the co-authors (BS) is the custodian of the laboratory information system, and all data were anonymised prior to extraction and analysis. We used the National Health Service (NHS) number as the primary identifier. Before this was available, patients were linked to their hospital number if they were known. Where neither of these were available, specimens were not extracted. Numerators were based on all tests, whether linked or not.

Recording of creatinine requests prior to 1993 was inconsistent and there were few records of ACR/PCR tests prior to 2006. In September 2009, the reference method for creatinine changed to isotope dilution mass spectrometry and all creatinine measurements have been adjusted to reflect this. For each test result, the sex and date of birth of the patient, and the date, location and name of the requesting physician were extracted from the database. A request was coded as coming from either primary care or other non-primary care (secondary or tertiary) care using an amended version of the in-house laboratory coding system. Locales that had requested less than 50 tests over the entire study period were not included in the analysis.

eGFR was calculated from SCr using the MDRD formula,^5^ chosen to reflect clinical practice in the timeframe of the study. We split eGFR into six categories of renal impairment using thresholds that define CKD. The first two represent normal (>90 mL/min/1.73 m^2^) and mildly impaired (60–89 mL/min/1.73 m^2^) kidney function and the remaining four represent moderate to severely impaired kidney function and correspond directly to CKD stages 3a (45–59 mL/min/1.73 m^2^), 3b (30–44 mL/min/1.73 m^2^), 4 (15–29 mL/min/1.73 m^2^) and 5 (<15 mL/min/1.73 m^2^) . The MDRD formula has an adjustment for ethnicity, which raises eGFR for non-white ethnicity. We were unable to obtain these data, so made no adjustment and hence our eGFR staging is biased slightly towards more severe renal impairment. HbA1c (expressed in International Federation of Clinical Chemistry and Laboratory Medicine (IFCC) units) was categorised into four levels: (1) not measured, (2) under the diagnostic thresholds for diabetes (<48 mmol/mol), (3) controlled glycaemia (48–58 mmol/mol) and (4) uncontrolled glycaemia (>58 mmol/mol). Albuminuria was categorised similarly using diagnostic thresholds as: (1) not measured, (2) measured but less than 3 mg/mol for ACR or less than 15 mg/mmol for PCR, (3) microalbuminuria (>3 mg/mol (ACR) and >15 mg/mmol (PCR)) and (4) macroalbuminuria (>30 mg/mmol (ACR) or >50 mg/mmol (PCR)).

### Number of creatinine and urinary albumin or protein tests over time

The total number of SCr, ACR and PCR tests ordered from primary care and non-primary care locales was calculated separately for each year from 1993 to 2013. In an additional analysis, we calculated age-adjusted yearly totals of creatinine testing in primary and secondary care by standardising to the age distribution in 1999. For this, we calculated creatinine testing rates per 5-year age brackets using population pyramid data for England between 1971 and 2011^13^ and estimates of the population of Oxfordshire local authority district^14^ as the denominator. These rates were multiplied by the reference age distribution and summed to form age-adjusted totals. Tests requested from primary care were additionally stratified by stages of renal impairment.

### Predictors of the frequency of monitoring

We examined factors related to the frequency of monitoring in a subcohort of people having kidney function/damage monitored, defined as having at least two tests further to the first year's measurement and complete covariate data. Measurements within the first year of follow-up were excluded as they are likely to be for reasons other than monitoring. We used Poisson regression to model the frequency of monitoring adjusting for initial level of kidney function, HbA1c testing, evidence of albuminuria or proteinuria, gender and age. All analyses were carried out using R V.3.1.2^15^

## Results

Data were obtained on 1 220 447 people, 527 753 of which had only one entry and the remaining 692 694 had median (IQR) follow-up of 7.6 (3.7–12.5) years. The percentage of specimens that could not be linked to either a hospital or NHS number was 20% in 1993 and dropped steadily to less than 1% in 2013 (see online supplementary figure S1).

### Number of kidney function tests over time

**Figure 1:**
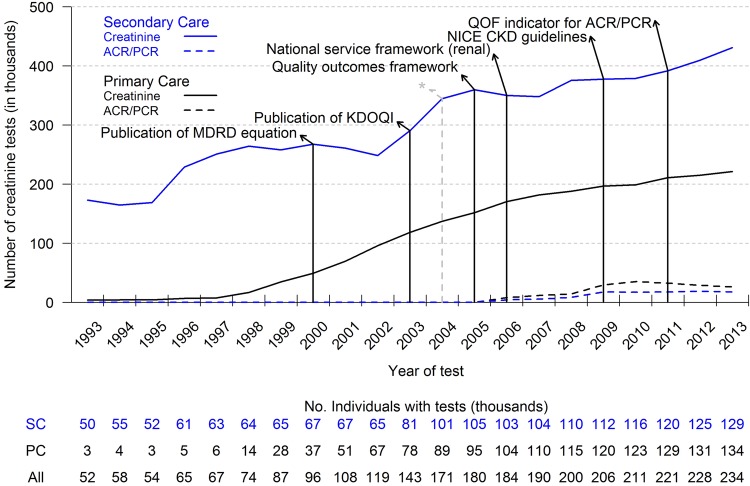
Total number of serum creatinine tests ordered from secondary and primary care between 1993 and 2013 and dates of key publications. *Indicates enlargement of population denominator. ACR, albumin-creatinine ratio; CKD, chronic kidney disease; KDOQI, Kidney Disease Outcomes and Quality Initiative; MDRD, Modification of Diet in Renal Disease; NICE, National Institute for Health and Care Excellence; PC, primary care; PCR, protein to creatinine ratio; QOF, Quality and Outcomes Framework; SC, secondary care.

Figure 1 shows the trend lines for both creatinine and ACR/PCR tests with dates of key publications, guidelines and changes to the QOF. Between 1993 and 2013, the last full year of follow-up, the number of creatinine requests from primary care locales increased from 4048/year to 221 557/year. Requests from secondary and tertiary care locales rose from 173 323 in 1993 to 431 198 in 2013. Record of requests for ACR/PCR tests began in 2006 and totalled 8125 in primary care and 4467 in secondary or tertiary care, and in 2013 the respective totals were 26 317 and 17 769.

The age distribution of people that have creatinine measured in Oxfordshire changed over the duration of the study. There were more people (as a proportion of the total) being tested in age brackets 85 or older in 2013, compared with 1999, but fewer in the 70–80s age brackets (see online supplementary figure S2). Adjusted creatinine totals were lower than unadjusted totals and suggest that for 2013, 11.9% of the tests in secondary care and 12.6% in primary care can be attributed to shifts in age demographics since 1999 (see online supplementary figure S3).

### Distribution of renal impairment over time in primary care

**Figure 2:**
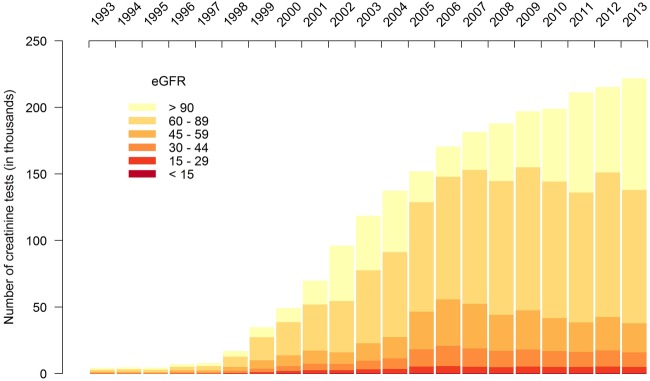
The number of serum creatinine tests between 1993 and 2013 by stages of renal impairment (eGFR, estimated glomerular filtration rate).

Figure 2 shows the total number of tests stratified by eGFR categories corresponding to the stages of CKD. Between 1998 and 2013, creatinine testing increased in primary care. The number of tests for normal, mildly reduced and impaired renal function (CKD stages 3–5) showed a marked increase between 1998 and 2005. However, after 2005, the number of tests showing impaired function remained stable. Therefore, all the increase after 2005 is in tests from patients with normal kidney function. The number of tests corresponding to CKD increased from 1648 tests in 1993 to 55 970 in 2006, but has since fallen back to 38 056 in 2013. Impaired kidney function tests accounted for 41% of all kidney function tests requested in 1993 and only 17% in 2013. A similar pattern was observed for secondary and tertiary care (figure not shown), and when limiting one test per patient (see online supplementary figure S4).

### Frequency of monitoring

There were 167 701 participants with at least two tests further to the first year's measurement and complete covariate data. Table 1 shows the results of the fitted model of monitoring frequency.

**Table 1:** Parameter estimates for the frequency of monitoring renal function in primary care

	n	Rate per year (95% CI)
Renal function as measured by eGFR (mL/min/1.73^2^)		
>60 (Reference)	146 604	1.09 (1.09 to 1.11)
45–59 (3a)	16 343	1.12 (1.11 to 1.12)
30–44 (3b)	3723	1.26 (1.25 to 1.27)
15–29 (4)	791	1.43 (1.40 to 1.47)
<15 (5)	240	1.28 (1.23 to 1.33)
Albuminuria		
Not measured	167 128	1
<3 mg/mmol	430	1.07 (1.03 to 1.12)
3–30 mg/mmol	116	1.06 (0.98 to 1.15)*
>30 mg/mmol	27	1.39 (1.19 to 1.60)
Time of initial measurement		
Pre-1998	38 679	1
1999–2003	63 530	1.06 (1.05 to 1.06)
2004–2008	51 998	1.16 (1.16 to 1.17)
2009–2013	13 494	1.88 (1.86 to 1.89)
Age (years)		
<20	9398	1
21–40	36 838	0.99 (0.98 to 1.00)*
41–60	61 106	1.20 (1.19 to 1.21)
61–80	51 405	1.45 (1.43 to 1.46)
81–100	8954	1.51 (1.49 to 1.53)
Not measured	105 770	1
HbA1c (mmol/mol)		
<48	49 570	1.11 (1.10 to 1.11)
48–58	5691	1.70 (1.69 to 1.71)
>58	6670	1.77 (1.77 to 1.79)
Sex		
Male	74 014	1
Female	93 687	0.98 (0.97 to 0.98)

*Significant at p<0.001 except.

eGFR, estimated glomerular filtration rate; HbA1c, glycated haemoglobin.

The average rate (95% CI) of monitoring in the reference group (males aged less than 20 with normal renal function, no albuminuria or diabetes) was 1.09 (1.09 to 1.11) tests per year (equivalent to one test every 335 days on average). Relative increases in the frequency of monitoring were found for older people, for people with microalbuminuria or macroalbuminuria, lower eGFR, those with any HbA1c tests and higher HbA1c. The frequency of monitoring was also shown to increase with time, independently of other associated factors. Female gender was associated with a small but statistically significant relative decrease in the frequency of monitoring. For a female patient aged between 61 and 80 with stage 3a CKD (eGFR 45–59 mL/min/1.73 m^2^), the average rate of monitoring is estimated as 3.23 tests per year with 95% CI (3.19 to 3.26) and for a similar woman with controlled glycaemia, the average number of tests rises to 5.50 tests per year on average with 95% CI (5.44 to 5.56).

## Conclusion

### Summary

We find that the number of SCr tests has risen dramatically since the late 1990s, especially in primary care, and exceeded 600 000 tests in 2013. Some of this increase can be attributed to a shift in the age distribution of the population and the expansion of the area that the laboratory serves in 2003. Publication of the KDOQI guidelines in 2002 came after the start of a sharp increase in test ordering from secondary or tertiary care doctors and later publications did not seem to influence test ordering rates. There was also little evidence that KDOQI, the introduction of QOF in 2004 and later extensions directly affected creatinine testing rates in primary care. In contrast, a rise in urinary ACR and PCR testing around 2009/2010 coincides with the introduction of relevant QOF indicators.

Older people, people with higher HbA1c and people with kidney disease were most frequently tested for SCr. Up until 2005, the increasing volume of testing in primary care was accompanied by increasing numbers with CKD among those tested. After 2005, the volume of creatinine testing continued to increase, but the number of tests with eGFR corresponding to CKD stages 3–5 stabilised or decreased while the number of eGFR tests with results in the normal or mildly impaired range (≥60 mL/min/1.73 m^2^) increased. The number of tests with results in the normal range (>90 mL/min/1.73 m^2^) has risen fastest since 2006/2007.

### Strengths and limitations

We had access to data from all requests over 20 years at a single laboratory that serves a well-defined population, typical of the wider UK population. However, we did not have data on patient history or prescriptions. In particular, we have no data on the reasons for why blood samples were taken for renal function testing. Given that the majority of laboratories will analyse sodium, potassium and creatinine as a set, the primary target of monitoring by a GP may be the electrolyte levels rather than the creatinine level. While this makes interpretation of the rise in creatinine testing more complex, if abnormalities are detected in electrolyte levels, this prompts an immediate clinical need to determine the creatinine level, and therefore creatinine testing is an important component of monitoring of electrolytes as well as for CKD itself. Furthermore, we cannot comment on whether tests were ordered with appropriate frequency, or to what extent the observed rise in testing reflects the increase in the UK in prescriptions for medicines, many of which require renal function testing.^4^ ^16^ Without ethnicity data, we were obliged to approximate eGFR by the MDRD equation without the appropriate adjustment for people of non-white ethnic group, and hence we underestimate eGFR and overestimate the amount CKD. However, this would affect fewer than 5% of people in Oxfordshire, and therefore we expect this would only make minor changes to the stage distributions in this sample.^17^

### Comparison with existing literature

To our knowledge, no other study has looked specifically at changes in SCr test ordering rates, over time, in the UK, but there are studies looking at CKD prevalence which have data on testing rates. One such study reported similar size increases in both the number of blood samples being taken and the number of people having their creatinine tested between 2004 and 2010.^18^ In contrast, an analysis of primary care computer records in Kent, Manchester and Surrey between 1998 and 2003, and a similar study in south-west London in 2007, each reported that 30% of patients had a valid SCr measurement.^19^ ^20^ The latter figures provide context for our analysis but are not directly comparable, since we do not have an equivalent denominator (total number of patients in the region served by the laboratory), and since these studies report number of patients rather than number of tests. A study using Health Survey for England data between 2003 and 2010 found a modest decline in rates of CKD, despite increases in diabetes and obesity.^21^ We have shown a similar decline in tests reflecting CKD in Oxfordshire in recent years, even after adjusting for risk factors such as age and HbA1c, but we have also shown that the total number of SCr tests ordered has continued to rise, driven by an increase in SCr tests with values above 90 mL/min/1.73 m^2^.

### Implications

The increase in the volume of kidney function tests in Oxfordshire, and in particular creatinine testing, is likely to be due to a number of factors. There is a net increase in number of people being tested year on year and people are being tested more frequently independent of changes in common risk factors for CKD. Given the uncertainty around the best methods and intervals for monitoring renal function, it is unclear whether more frequent monitoring may lead to a net benefit, but as eGFR has considerable biological variability,^22^ more frequent monitoring may lead to many false alarms and overadjustment of treatment.^23^ Conversely, increased laboratory workloads and the subsequent strain on limited resources may contribute to an increase in missed or delayed diagnoses.^24^ If resources allowed, electronic health records could be used to identify abnormal creatinine tests for further investigation, potentially reducing delays.^25^ However, it is not currently clear whether increased test ordering in the UK contributes to errors in the management of CKD, or leads to clinically relevant delays in diagnosis (see online supplementary figure S5). Since most of the recent increase in testing is driven by tests on patients with normal to mildly reduced eGFR and as there is little evidence of benefit from intervening in people with early stage CKD,^26^ these results are unlikely to influence clinical decisions or contribute to better care. Future work using a richer data source with prescription records and GPs’ notes could help unravel the underlying reasons for the increased testing and determine how much is necessary and useful.
